# Oxidized Mutant Human Hemoglobins S and E Induce Oxidative Stress and Bioenergetic Dysfunction in Human Pulmonary Endothelial Cells

**DOI:** 10.3389/fphys.2017.01082

**Published:** 2017-12-19

**Authors:** Sirsendu Jana, Fantao Meng, Rhoda E. Hirsch, Joel M. Friedman, Abdu I. Alayash

**Affiliations:** ^1^Laboratory of Biochemistry and Vascular Biology, Center for Biologics Evaluation and Research, Food and Drug Administration, Silver Spring, MD, United States; ^2^Hematology Division, Department of Medicine and Department of Anatomy and Structural Biology, Albert Einstein College of Medicine, Bronx, NY, United States; ^3^Department of Physiology and Biophysics, Albert Einstein College of Medicine, Bronx, NY, United States

**Keywords:** mutant hemoglobins, hemoglobin S, hemoglobin E, ferryl hemoglobin, pulmonary endothelial cells

## Abstract

Cell free hemoglobin (Hb), becomes oxidized in the circulation during hemolytic episodes in sickle cell disease (SCD) or thalassemia and may potentially cause major complications that are damaging to the vascular system. Hemolytic anemias are commonly associated with pulmonary hypertension (PH) and often result from dysfunction of lung endothelial cells. The aim of this study was to determine the effect of different Hbs on cultured human lung endothelial function. Toward this goal, endothelial permeability, oxidative stress response parameters, glycolytic and mitochondrial bioenergetic functions were monitored in cultured human pulmonary arterial endothelial cells (HPAEC) following incubation with human adult Hb (HbA), and Hb isolated from patients with sickle cell Hb (HbS, βV6E) and HbE (βE26K) that commonly co-exist with β-thalassemia. These mutant Hbs are known for their distinct oxidative profiles. HPAEC treated with the ferrous forms of HbE, HbS for 24 h showed higher loss of endothelial monolayer integrity with concomitant rise in reactive oxygen radical production, lipid hydroperoxide formation and higher expressions of oxidative stress response proteins including heme oxygenase-1 (HO-1) accompanied by a rise in uncoupled mitochondrial respiration. Loss of membrane permeability was diminished in part by haptoglobin (Hp, protein scavenger), hemopexin (Hpx, heme scavenger) or ascorbate (reducing agent). To understand the role of Hb oxidation, HPAEC were exposed to ferric or ferryl states of the mutant Hbs. Ferryl forms of all proteins caused a significant damage to the endothelial monolayer integrity at a higher degree than their respective ferric Hbs. Ferryl forms of HbS and HbE also caused a loss of respiratory chain complex activities in isolated endothelial mitochondria and basal oxygen consumption in HPAEC. However, longer incubation with ferryl Hbs produced bioenergetic reprogramming including higher degree of uncoupled respiration and glycolytic rate. The data in this report collectively indicate that higher oxidation forms of HbS and HbE cause endothelial dysfunction through distinct damaging mechanisms involving mitochondrial bioenergetic function.

## Introduction

Hb constitutes almost 95% of all the proteins within red blood cells (RBC); it is a tetramer of two α and two β subunits (α2β2), with a heme (Fe^+2^) moiety bound to each subunit. Hemolysis or rupture of RBCs occur in various disease conditions and most importantly in hemoglobinopathies including SCD and β-thalassemia leading to the release of free Hb molecules to the circulation (Buehler et al., 2011; Schaer et al., 2013). The Hb molecule is highly susceptible to heme/iron oxidation but the highly reducing environment within RBCs (due to the presence of antioxidant enzymes like catalase, superoxide dismutase (SOD) and methemoglobin reductase) prevents the oxidation of Hb and maintains it in the functional ferrous form (HbFe^2+^). Cell free Hb in circulation undergoes oxidation to produce its primary oxidized derivative, ferric Hb (HbFe^3+^) that dissociates into α-β dimers. At the tissue level ferric or ferrous Hb can also form highly reactive ferryl species (HbFe^4+^) upon reacting with locally produced hydrogen peroxide (H_2_O_2_) or its own synthesized H_2_O_2_ in a pseudoperoxidase cycle (Alayash, 2014). The cycle consists of three distinct steps: (1) initial oxidation of HbFe^2+^ to HbFe^4+^, (2) autoreduction of the HbFe^4+^ intermediate to HbFe^3+^, and (3) reaction of ferric/metHb with an additional H_2_O_2_ molecule to regenerate the ferryl intermediate/ferryl protein radical (·HbFe^4+^ = O) (Alayash, 2004, 2014). This radical may migrate to the oxidation “hotspot” to further damage the protein, including the irreversible oxidation of βCys93 and subsequent dimerization of the protein (Alayash, 2004). Because this surface amino acid is an important endpoint for free radical induced oxidation (Jia et al., 2007; Pimenova et al., 2010) our group has developed βCys93 as a quantitative reporter for H_2_O_2_ induced oxidation (Kassa et al., 2017; Strader and Alayash, 2017). Both ferric and ferryl species are known to release highly toxic heme moieties in the circulation from the parent Hb molecule (Kassa et al., 2016). These oxidized species of Hb and heme may cause vascular complications involving inflammation, vasoconstriction and endothelial dysfunction. Dysfunction of lung endothelial cells leading to pulmonary arterial hypertension (PAH) has increasingly been recognized in severe β-thalassemic or SCD patients and in other hemolytic conditions (Gladwin et al., 2010; Morris and Vichinsky, 2010).

Heme resulting from Hb oxidation has been shown to act as a damage-associated molecular pattern (DAMP) molecule that can activate Toll-like receptor-4 (TLR4) of the innate immune system leading to oxidant production, inflammation and vascular injury (Gladwin et al., 2010; Belcher et al., 2014). Several other mechanisms, e.g., nitric oxide (NO) sequestration by Hb, chronic inflammatory reactions triggered by activation of NF-κB transcription factor and the hypoxia inducible factor (HIF-1α), have all been also proposed to contribute to the endothelial dysfunction mediated by cell free Hb proteins (Manalo et al., 2008). Although endothelial cells are relatively more dependent on glycolysis for their ATP source, growing numbers of experiments have highlighted the importance of mitochondrial bioenergetics and signal-modulatory roles played by mitochondria in the maintenance of endothelial health that ultimately contributes to normal vascular function (Davidson and Duchen, 2007). Because mitochondria are one of the most critical regulators in cell survival, recently various functional mitochondrial bioenergetic parameters have been utilized for the assessment of human bioenergetic health index (BHI) (Chacko et al., 2014). In this context, the effects of cell free Hb on cellular functions, mitochondrial bioenergetics have not been fully explored. We and others have recently shown the potential of Hb molecules particularly in their oxidized forms promoting various degrees of bioenergetic impairment in cultured cells and in platelets (Higdon et al., 2012; Cardenes et al., 2014; Kassa et al., 2015; Chintagari et al., 2016). Using mouse lung epithelial cells (E10), we specifically showed differential toxicity of the oxidized ferric and ferryl species of HbA and HbS affecting some key signaling pathways and bioenergetic functions through oxidative insults to those cells (Kassa et al., 2015; Chintagari et al., 2016).

It is known that plasma levels of Hb in thalassemic patients, due to hemolysis, can increase significantly, and in sickle cell disease it can reach as high as 25 μM in patients experiencing typical sickle cell crises (Schaer et al., 2013). Moreover, as we have recently shown, both HbE and HbS exhibit defective pseudoperoxidase activities resulting in accumulation of higher levels of ferryl Hb in solutions (Kassa et al., 2015; Strader et al., 2016).

Since, several toxicity studies emphasizing the effect of Hb on endothelial function have indicated the possible involvement of oxidative reactions triggering the activation of inflammatory and death pathways we thought it would be interesting to explore the toxicity of different naturally occurring Hb variants with distinct oxidative profiles on pulmonary endothelial cells in the context of PAH. In this investigation, we focused our efforts on the impact of Hb oxidative pathways on endothelial functions and modulation of metabolic responses using different naturally occurring Hb variants with distinct oxidative profiles in the context of PAH. Toward this goal we used human pulmonary artery endothelial cells (HPAEC) and monitored endothelial permeability, oxidative stress response parameters following exposure to either HbA or HbS (βV6E) or HbE (βE26K). Simultaneously, for the first time to the best of our knowledge, we also compared the changes in mitochondrial bioenergetics and glycolytic rate in HPAECs exposed to these different Hbs by measuring the oxygen consumption rate (OCR) and extracellular acidification rates (ECAR), respectively using an extracellular flux analyzer. Further, we showed that the exposure of HPAEC to the ferryl forms of these mutant Hbs resulted in impairments of electron transport chain complex function, specifically complex IV activity in isolated endothelial mitochondria. Therefore, this work establishes a link between the loss of endothelial permeability and bioenergetic impairment conferred by oxidized forms of naturally occurring and mutant cell free Hbs.

## Materials and methods

### Preparation of various hemoglobins (HbA, HbE, and HbS)

Blood was obtained from normal and SCD patients attending the National Institutes of Health, Bethesda, Maryland with informed consent. Hemoglobin E was purified from red blood cells obtained from transgenic mice expressing solely human HbE as described earlier (Chen et al., 2012). Hb (HbS/HbE/HbA) was purified from erythrocyte lysates from all sources by anion DEAE and cation CM chromatography respectively (Manjula and Acharya, 2003). Red blood cells were washed three times with PBS and centrifuged at 500 g for 20 min using a Legend X1R centrifuge (Thermo Scientific). RBCs were lysed with 3-fold volumes of water and mixed gently with a glass rod at room temperature for 30 min. Final salt concentration was adjusted to about 0.9% with NaCl. The lysate was then centrifuged at 12,000 g for 40 mins. The supernatant was filtered with 0.2 μM membranes, and then dialyzed against 50 mM Tris-acetate buffer, pH 8.3, at 4°C.

The lysate was loaded onto a DEAE Sepharose Fast Flow column, which was equilibrated with 6 column volumes of 50 mM Tris-acetate at pH 8.3 (buffer A) using an AKTA FPLC System. Hb was eluted at 4°C with a linear gradient of 25–100% buffer B, 50 mM Tris-acetate at pH 7.0 in 12 column volumes. The column was eluted at a flow rate of 2 ml/min and the effluent was monitored at 540 and 630 nm. Hb was collected, concentrated, and then dialyzed against extensive 10 mM phosphate buffer (buffer C), pH 6.5, at 4°C. The Hb solution was then applied to a CM Sepharose Fast Flow column, which was equilibrated with 6 column volumes of buffer C using an AKTA FPLC system. Hb was eluted (at 4°C) with a linear gradient of 0-100% buffer D, 15 mM phosphate buffer (at pH 8.5) in 12 column volume (2 mL/min) and the effluent was monitored at 540 and 630 nm. Hb was collected, concentrated, dialyzed against PBS, and was stored at −80°C for future use. Purified Hb in PBS solutions was then dialyzed against water for 12 h at 4°C for 3 times. The complete removal of antioxidative enzymes, namely SOD and catalase in the purified Hb solutions was verified as previously reported (Aebi, 1984). The criteria of purity of three proteins were verified by isoelectric focusing and HPLC. Molar concentrations for all Hb solutions in this paper are calculated and based on heme (Meng and Alayash, 2017).

### Preparation of ferric and ferryl hemoglobins

The ferric (met) Hb was generated as previously reported (Meng and Alayash, 2017) by incubating 2 mM ferrous (oxy) Hb with 3 mM potassium ferricyanide in PBS at room temperature for 15 min. Excess ferro- and ferricyanide were removed by passing the reaction solution through a Sephadex G-25 column equilibrated with PBS at 4°C. Ferryl Hb solutions were prepared as previously reported by mixing metHb (1 mM) with H_2_O_2_ (20 mM) in PBS buffer at room temperature for 1 min, and then the excess H_2_O_2_ was rapidly removed by passing the reaction solution through two G25 desalting columns (HitrapTM, 5 mL, GE Healthcare) connected in series (Kassa et al., 2015).

### Endothelial cell culture

Cryopreserved human pulmonary artery endothelial cells (HPAEC) were purchased from Life Technologies Corporation (Thermo Fisher Scientific, Waltham, MA) and were cultured in Medium 200 supplemented with Low Serum Growth Supplement (LSGS) containing FBS (Thermo Fisher Scientific, Waltham, MA). Some HPAEC were also cryopreserved per the manufacturer's protocol within passage 4. The cells were used between passages 6–10 after thawing. The media was changed every 48 h. Cells were subcultured after attaining 80% confluency.

### Exposure of HPAEC to hemoglobins

For all experiments HPAEC were grown to 80–90% confluency in complete media. Before any treatment, cells were serum starved for 6 h in a medium composed of Medium 200 supplemented with all the components of LSGS Kit except FBS (Thermo Fisher Scientific, Waltham, MA). The cells were then exposed to ferrous (HbFe^2+^), ferric (HbFe^3+^) or ferryl (HbFe^4+^) for varying periods of time up to 12 h. Antioxidants, scavengers or other protective agents were added to the media prior to the addition of Hb. In most of the experiments HPAEC were washed three times with either pre-warmed phosphate buffered saline (PBS) or with complete media to remove Hb proteins and other additions otherwise mentioned. Following exposure to specified time periods, cell lysates were prepared for further studies.

### Endothelial permeability study

For permeability determinations, HPAEC were grown to confluence on collagen coated PTFE-Transwell membrane inserts (Corning Inc. Corning, NY) placed on a 24-well culture plate forming a two-compartment system. Approximately, 2 × 10^5^ cells in 0.1 ml of complete medium were seeded onto the membrane insert with pore size of 0.4 μm following a previously published method (Nooteboom et al., 2000). The lower compartment was then supplemented with 0.6 ml of complete media to equilibrate the hydrostatic fluid pressures across the membranes per the manufacturer's protocol. Cells were grown up to 5–6 days until they form a tight confluent monolayer.

To determine the macromolecular passage across the membranes, 0.1 ml of FITC-labeled dextran, 40 KD (200 μg/ml) solution prepared in complete media with or without Hbs and other additives was added onto the membrane insert following complete removal of culture medium. Simultaneously each well in the lower chamber was replenished with 0.6 ml of fresh culture medium. Diffusion of dextran was monitored after varying periods of incubation by measuring the FITC-green fluorescence in the medium in the lower chamber using a Synergy HTX Multi-Mode Reader (Biotek Instruments, Inc., Winooski, VT).

### Immunoblotting and microscopy

HPAEC were rinsed in phosphate-buffered saline following incubation and lysed in RIPA buffer containing protease inhibitor to prepare whole cell lysate. Cell lysate proteins were separated by electrophoresis using precast 4–20% NuPAGE bis-tris gels (Thermo Fisher Scientific, Waltham, MA) and then transferred to nitrocellulose membranes (BioRad, Hercules, CA). Membranes were blocked in 5% milk-PBS and then processed for Western analysis using specific primary antibodies as described in the figure legends. Mouse monoclonal anti-hemoxygenase-1, rabbit polyclonal anti-H-ferritin antibodies were purchased from Abcam (Cambridge, MA). Rabbit polyclonal phospho-NF-κB p65, rabbit polyclonal NF-κB p65 were purchased from Cell Signaling Technology (Danvers, MA).

To detect DMPO-nitrone adduct formation in Hb treated HPAEC; cells grown on coverslips were treated with various Hb species for 2 h. Cells were thoroughly washed in PBS and then treated with 1 mM DMPO for 30 min in the culture media. Cells were then fixed in 4% paraformaldehyde after three consecutive wash with PBS. Immunocytochemistry was done as described earlier (Kassa et al., 2015) following incubation with polyclonal primary antibody against DMPO-nitrone adduct, (Cayman Chemical) at 4°C overnight. Alexa Fluor-594 conjugated goat anti-rabbit secondary antibody was used to visualize the cells under EVOS epifluorescence microscope (Thermo Fisher Scientific, Waltham, MA).

### Cellular oxidative stress parameters

Following treatment with various Hb species, endothelial lipid hydroperoxide levels were measured in HPAEC using a commercially available kit (Cayman Chemical Co., Ann Arbor, MI). Mitochondrial superoxide generation in HPAEC was monitored by a Synergy HTX multimode plate reader (Biotek Instruments, Inc., Winooski, VT) at 580 nm by labeling the cells with MitoSOX red dye (5 μM) (Thermo Fisher Scientific, Waltham, MA) for 30 min following incubation with a specific Hb species.

### Mitochondrial bioenergetic and glycolytic flux measurements

Mitochondrial bioenergetic function and glycolytic flux were simultaneously monitored in HPAEC using the XF24 extracellular flux analyzer (Seahorse Bioscience, Billerica, MA) as described before (Kassa et al., 2016). Briefly, HPAEC were seeded (100,000 cells/well) and cultured for 24 h in 24-well XF-cell culture plate (V7) obtained from Seahorse Bioscience and pre-coated with collagen I (Thermo Fisher Scientific, Waltham, MA). Prior to exposure, media was changed with serum-free media for 6 h. Cells were exposed to various Hb proteins for up to 24 h. Mitochondrial OCR was assessed after washing the Hb containing media with XF-assay media (Seahorse Bioscience, Billerica, MA) supplemented with 10 mM glucose, 5 mM pyruvate, and 2 mM glutamate as per the manufacturer's instructions. The XF-assay media used for measuring the extracellular acidification rate (ECAR) was free of glucose. For some experiments, Hb solutions were directly injected onto cells through the automated injection ports; and OCR/ECAR and were monitored up to 2 h prior to other additions. To obtain OCR profile, a sequential injection of oligomycin (1 μM), carbonyl cyanide-p-trifluoro-methoxyphenylhydrazone (FCCP, 1 μM) and a combination of rotenone and antimycin A (1 μM each) were made following manufacturer recommended protocol (Seahorse Bioscience, Billerica, MA). Similarly, to obtain ECAR profile glucose (10 mM), oligomycin (1 μM) and glycolytic inhibitor 2-deoxyglucose (2-DG, 100 mM) were sequentially added to the wells through automated injections. The values from individual wells were recorded and plotted using XF24 software, version 1.8. Blank wells with Hb variants were also run to eliminate any background OCR. However, Hb species showed no noticeable interference on either OCR or ECAR. Various cellular bioenergetic parameters e.g., basal and maximal respiration were calculated as described earlier (Kassa et al., 2015). Glycolytic parameters e.g., basal glycolysis, glycolytic capacities were calculated following a previously published method (TeSlaa and Teitell, 2014).

### Isolation of mitochondria

Mitochondrial fractions were isolated from HPAEC cells following the manufacturer's protocol, using the mitochondria isolation kit designed for cultured cells (Pierce Biotechnology, Rockford, IL, USA). The final mitochondrial pellet was resuspended in an isotonic buffer containing 145 mM KCl, 50 mM sucrose, 5 mM NaCl, 1 mM EGTA, 1 mM magnesium chloride, 10 mM phosphate buffer, pH 7.4 and stored at −80°C for further use.

### Mitochondrial respiratory chain complex activity

Frozen and thawed samples of mitochondria were incubated for 2 h at 37°C in the presence or absence of the Hb proteins in a total volume of 200 μl. Following incubation, mitochondria were thoroughly washed by centrifugation at 4°C with an excess of ice-cold 50 mM phosphate, pH7.4 to ensure complete removal of the residual Hb proteins. Interference of any residual Hb bound to mitochondria was monitored by using appropriate blanks and blank values were subsequently subtracted. An aliquot of the mitochondrial suspension (20 μg of protein) was utilized to measure complex I or complex IV activities. Complex I activity was assayed by using ferricyanide as the electron acceptor (Hatefi, 1978; Khan et al., 2005). The assay was carried out at 30°C in a reaction system containing 0.17 mM NADH, 0.6 mM potassium ferricyanide, 0.1% (v/v) Triton-X 100 in 50 mM phosphate buffer, pH 7.4. The rate of oxidation of NADH was monitored by the decrease in absorbance at 340 nm after the addition of mitochondrial suspension to the sample cuvette (Khan et al., 2005).

Succinate supported reduction of ferricytochrome c to ferrocytochrome c at 550 nm was monitored to measure the activity of mitochondrial complex II–III (succinate–cytochrome c reductase) following the method by Jana et al. (2011). The reaction was initiated by adding the mitochondrial suspension (20 μg) to the sample cuvette. The absorbance change at 550 nm was monitored for a period of 3 min. The assay was repeated with antimycin A (10 μM) and the enzyme activity was calculated by subtracting the antimycin A sensitive rate from overall rate and expressed as μmoles oxidized cytochrome c reduced/min/mg protein (Jana et al., 2011).

The activity of complex IV was assayed following the oxidation of reduced cytochrome c (ferrocytochrome c) at 550 nm in 10 mM phosphate buffer, pH 7.4 at room temperature (Khan et al., 2005). K-ferricyanide (1 mM) was added to oxidize ferrocytochrome c in the blank cuvette and the reaction was initiated in the sample cuvette by the addition of mitochondrial suspension. The activity of the enzyme was calculated from the first order rate constant and the concentration of reduced cytochrome c in the sample cuvette as published earlier (Khan et al., 2005).

### Statistical analysis

All values are expressed as mean ± SEM. Values from two treatment groups were compared using non-parametric Mann-Whitney *U*-test (Altman, 1991). Data obtained from bioenergetic experiments using extracellular flux analyzer were compared using paired Student's *t*-test following previously published work (Kassa et al., 2015). A *p*-value of < 0.05 was considered as statistically significant.

## Results

### Autooxidation kinetics of hemoglobin variants and heme release

To illustrate the differences in oxidative profiles of the Hbs included in this study, Table 1 summarizes previously published autoxidation kinetics of the ferrous as well as heme loss from the ferric forms of HbE and HbS. These autoxidation rates are compared with those derived for human HbA. Also, included in this table are the oxygen affinity parameters of Hbs in solutions under normal experimental conditions. P_50_ values for HbA and HbE are reported in Table 1 and show little difference as reported earlier (Bunn et al., 1972; Gacon et al., 1974). The initial rate of autoxidation (k_auto_) for HbE is close to that of Human HbA, but as can be seen HbS undergoes a slightly higher rate of oxidation than HbA and HbE respectively. Heme loss from the ferric forms from both mutant Hbs run at much higher rates than that of normal HbA. Both HbE and HbS were shown to have unique pseudoperoxidative pathways that set them apart from normal HbA (Kassa et al., 2015; Strader et al., 2016).

**Table 1:** Oxygen equilibrium parameters, autooxidation and heme loss kinetics of mutant hemoglobins (HbE and HbS) contrasted with values for normal human hemoglobin.

**Sample**	**P_50_**	**n_50_**	**k_auto_**	**K_auto_ + Cat**	**Heme loss**	
	**mm Hg**		**h^−1^**	**h^−1^**	***k*_1_ (h^−1^)**	***k*_2_ (h^−1^)**
HbA	14.0^a^	2.6^b^	0.054 ± 0.002^b^	0.007 ± 0.003^b^	11.75^b^	0.86^b^
HbE	12.4^c^	2.8^c^	0.051 ± 0.054^b^	0.089 ± 0.002^b^	19.07^b^	2.12^b^
HbS	35.5^e^	1.75^e^	0.177 ± 0.042^d^	0.073 ± 0.016^d^	15.5^d^	1.68^d^

a *Strader et al. (2016)*,

b *Strader et al. (2017)*,

c *May and Huehns (1975)*,

d *Kassa et al. (2015)*,

e *Kassa et al. (2017)*.

### Hemoglobin variants cause endothelial oxidative stress

**Figure 1:**
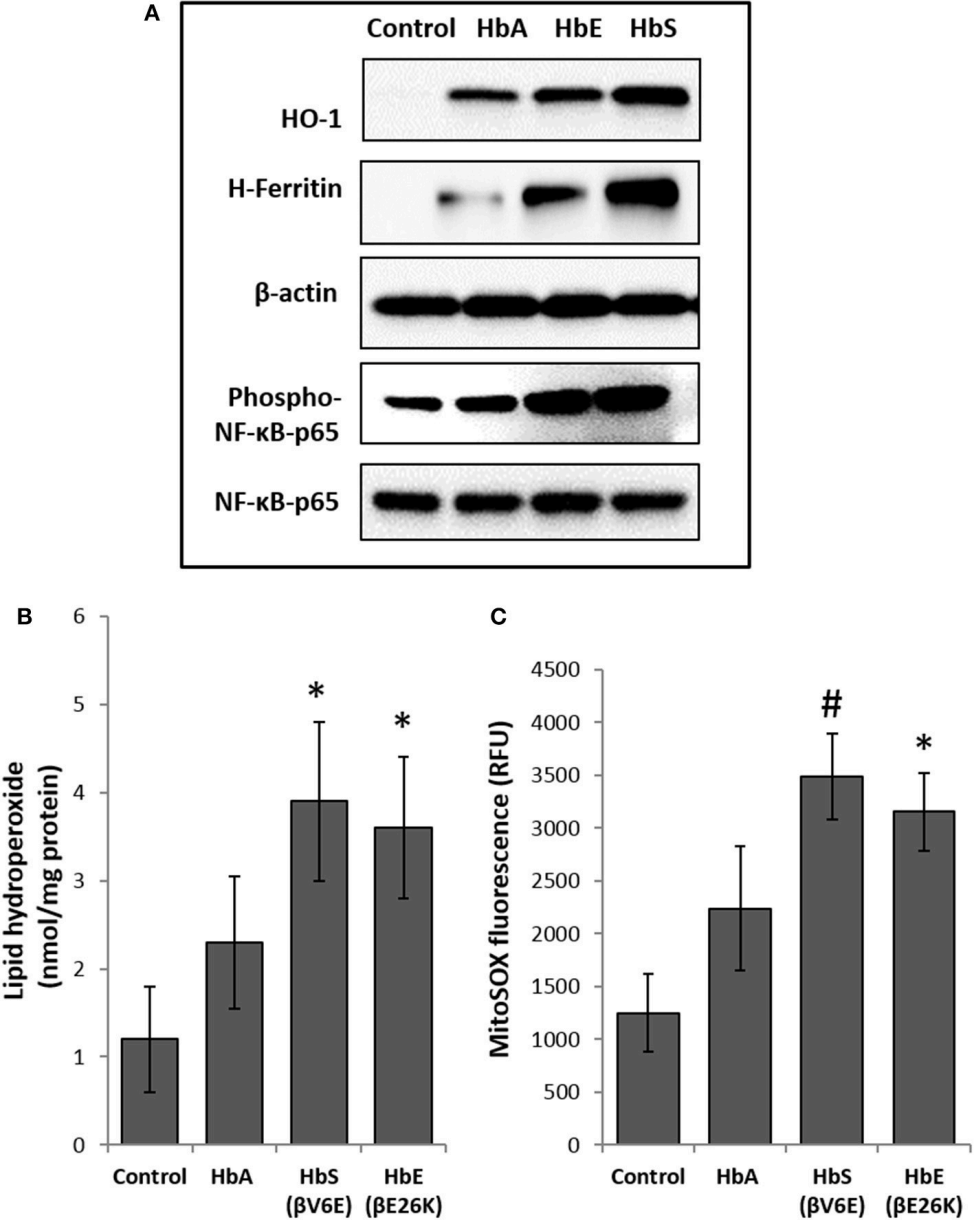
Effect of normal and mutant human hemoglobins on human pulmonary arterial endothelial cells (HPAEC). **(A)** HPAEC were exposed to ferrous forms of either HbA, HbS, or HbE at equimolar concentration (100 μM) for 12 h. Cell lysates were immunoblotted with primary antibodies against HO-1, H-ferritin, phospho-NF-κB-p65 and NF-κB-p65 proteins. Equal loading was confirmed by re-probing the blots against β-actin. Blots are representative of three independent experiments. **(B)** Levels of lipid hydroperoxides and **(C)** mitochondrial superoxide radical generation was measured in HPAEC following exposure to different Hb proteins as described in the methods section (*N* = 3). Values from different treatment groups were compared using Mann-Whitney *U*-test. ^*^P < 0.05 vs. untreated control, ^#^*P* < 0.001 vs. untreated control.

To explore cellular stress signaling in pulmonary endothelial cells, we first incubated Hb variants at equimolar concentration (100 μM/heme) with cultured HPAECs for up to 12 h and monitored the expression of HO-1 and H-ferritin protein levels in in these cells. HO-1 and H-ferritin both are strong indicators of oxidative stress as a result of heme release and subsequent iron load within the cellular compartment. Exposure to HbA and mutants HbE (βE26K) and HbS (βV6E) caused a robust increase in both HO-1 and H-ferritin expression (Figure 1A). In fact, at equimolar concentrations both HbE and HbS treated HPAECs showed significantly higher HO-1 and H-ferritin than the corresponding HbA. To monitor any upstream signaling event, we also probed the immunoblotted proteins from Hb-treated cell lysates for phosphorylated NF-κB p65 subunit. Both HbE and HbS treatment caused higher levels of phosphorylation in NF-κB p65 subunit as seen in Figure 1A.

To assess the extent of oxidative toxicity in various Hb exposed HPAEC, we measured lipid hydroperoxide formation following 12 h of incubation. Consistent with our results on HO-1 and H-ferritin expressions, all the Hb proteins caused a significant accumulation of lipid-hydroperoxides in HPAEC membrane lipid extracts. However, compared to HbA, levels of lipid hydroperoxides were substantially higher in both HbE and HbS treated cells (Figure 1B).

Our previous studies on the effects of normal human Hb on lung epithelial cells and other cell lines have shown a link between HO-1 expression and mitochondrial functional alteration (Converso et al., 2006; Bindu et al., 2015; Kassa et al., 2015; Chintagari et al., 2016). Since, (1) mitochondria are the most potent intra-cellular source of reactive oxygen species (ROS), and (2) to correlate our results with HO-1 expression, we first monitored mitochondrial superoxide generation as an indicator of electron flow impairment through the mitochondrial respiratory complexes by using the fluorescent MitoSOX-probe. As expected, both HbE and HbS caused significant increase in MitoSOX fluorescence in HPAEC (Figure 1C).

**Figure 2:**
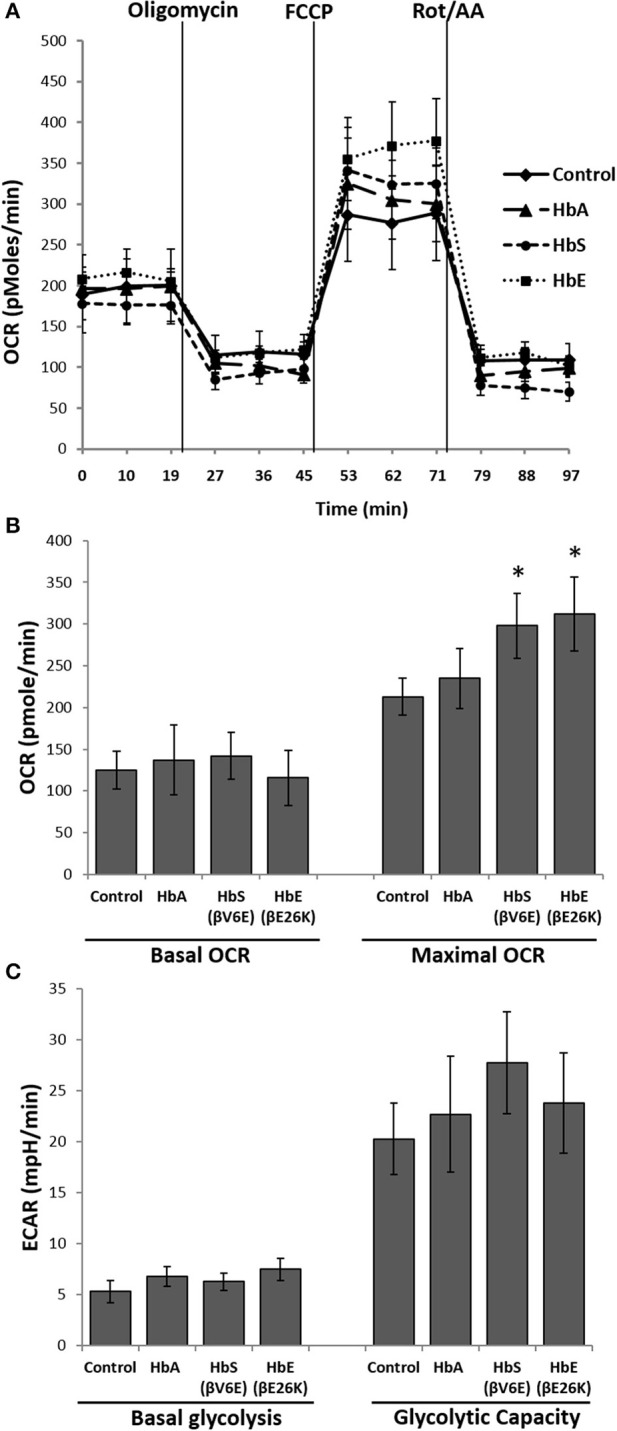
Effect of mutant hemoglobins on pulmonary endothelial bioenergetics. HPAEC were exposed to ferrous forms of HbA, HbS, or HbE at equimolar concentration (100 μM) for 12 h. Mitochondrial oxygen consumption rates (OCR) and extracellular acidification rates (ECAR) were measured in real time using XF24 extracellular flux analyzer. **(A)** A representative OCR plot showing the components of mitochondrial bioenergetic parameters. Average OCR values from individual set of experiments repeated four times were plotted to make bioenergetic profile of each Hb treated HPAEC. **(B)** Basal and maximal OCR obtained from HPAEC following exposure to Hb (*N* = 4). **(C)** Basal glycolysis rate and glycolytic capacity were calculated from HPAEC following exposure to Hb (*N* = 3). Differences between two different groups were compared using a paired Student's *t*-test. ^*^P < 0.05 vs. control.

Next we used the extracellular flux analyzer (XF Assay) to assess the energy utilization in HPAEC exposed to Hbs by monitoring mitochondrial bioenergetics and glycolytic proton flux in real time. oxygen consumption rate (OCR) and extra-cellular acidification rate (ECAR) in HPAEC were obtained from the XF assays as indicators of mitochondrial respiration and cellular glycolytic activity respectively. Exposure to HbA did not cause any significant changes in either basal OCR or maximal OCR obtained after FCCP injection in the HPAEC (Figures 2A,B). However, both HbE and HbS treated cells showed a higher rate of oxygen consumption following FCCP addition, indicating a mild uncoupling effect by those two Hb variants. Although, HbE and HbS treated HPAECs had no difference in the basal OCR (Figures 2A,B). We also measured glycolytic rates in HPAEC under the influence of Hb variants under similar experimental conditions. Basal glycolysis and glycolytic capacity were mostly unaffected by Hb variants as shown in Figure 2C.

### Effect of hemoglobin variants on pulmonary endothelial permeability

**Figure 3:**
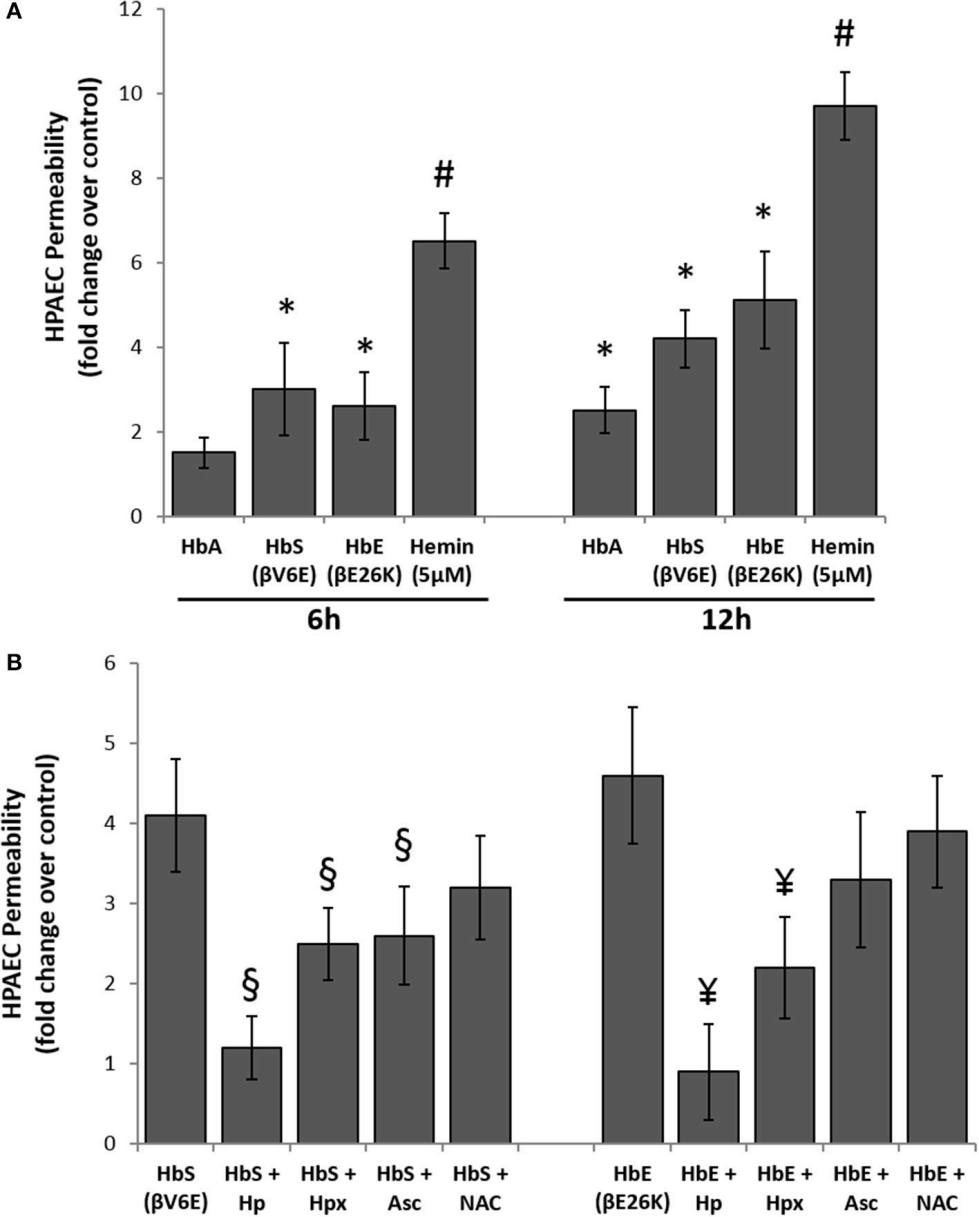
Cell free hemoglobin variants causes disruption of pulmonary endothelial monolayer. **(A)** HPAEC monolayer were grown on Transwell inserts and then exposed to ferrous forms of HbA or HbS or HbE at equimolar concentration (100 μM) either for 6 or 12 h in the presence of FITC conjugated dextran, 40 KD. Endothelial monolayer integrity was assessed by measuring the passage of FITC-dextran molecule through monolayer. FITC-green fluorescence was monitored by fluorescence plate reader as described in the methods section. Values are presented as average fold difference over control (*N* = 3). **(B)** In a similar set of experiments HPAEC were exposed to either HbS (100 μM) or HbE (100 μM) with or without equimolar of either Hp, Hpx or Asc or NAC for 12 h in the presence of FITC conjugated dextran. Endothelial monolayer integrity was assessed as mentioned before by measuring FITC-dextran fluorescence in the bottom chamber (*N* = 3). Values obtained from different treatment groups were compared using Mann-Whitney *U*-test. ^*^P < 0.05 vs. untreated control, ^#^*P* < 0.001 vs. untreated control, ^§^*P* < 0.05 vs. HbS, ^¥^*P* < 0.05 vs. HbE.

To explore the effects of Hb oxidation intermediates and toxicity on pulmonary endothelial cells, we first incubated different naturally occurring Hb variants at equimolar concentration (100 μM/heme) with cultured HPAECs grown on a tight monolayer on specially designed Transwell inserts for up to 12 h, and then observed endothelial permeability by measuring the passage of FITC conjugated-dextran (40 KD) through the monolayers (Nooteboom et al., 2000). The ferrous form of normal Hb (HbA) did not cause any significant changes in permeability within the first 6 h, however, we found higher but significant dextran passage after 12 h (Figure 3A). In contrast, both ferrous Hbs i.e., HbE (βE26K) and HbS (βV6E) caused significant changes in dextran permeability of HPAEC monolayers within 6 h (Figure 3A). The impairment of barrier function by both HbE and HbS was more pronounced after 12 h. However, hemin caused a robust change in dextran-permeability in those cells when used at a low concentration (5 μM) compared to the Hb proteins, which suggests that heme released from Hb as result of oxidation of the protein may play a key role in genesis of oxidative stress.

To understand the oxidative mechanism behind the impairment of permeability function by both HbE and HbS, we introduced several scavengers and antioxidants, e.g., haptoglobin (Hp), hemopexin (Hpx), antioxidant ascorbate (Asc), and thiol antioxidant N-acetyl cysteine (NAC) to the medium. Hp, a scavenger of Hb almost completely prevented the loss of barrier functions by either HbE or HbS when added at equimolar concentration (100 μM) (Figure 3B). On the other hand, only a partial protection (~40–50%) was achieved by co-incubation with 100 μM of the heme scavenger Hpx. Further, Asc (100 μM) provided partial but significant protection against HbE or HbS mediated permeability changes in the cultured HPAECs indicative of an underlying oxidative mechanism driving these processes (Figure 3B). However, NAC failed to confer any protection against HbS or HbE (Figure 3B).

### Effect of oxidized hemoglobin variants on pulmonary endothelial permeability

**Figure 4:**
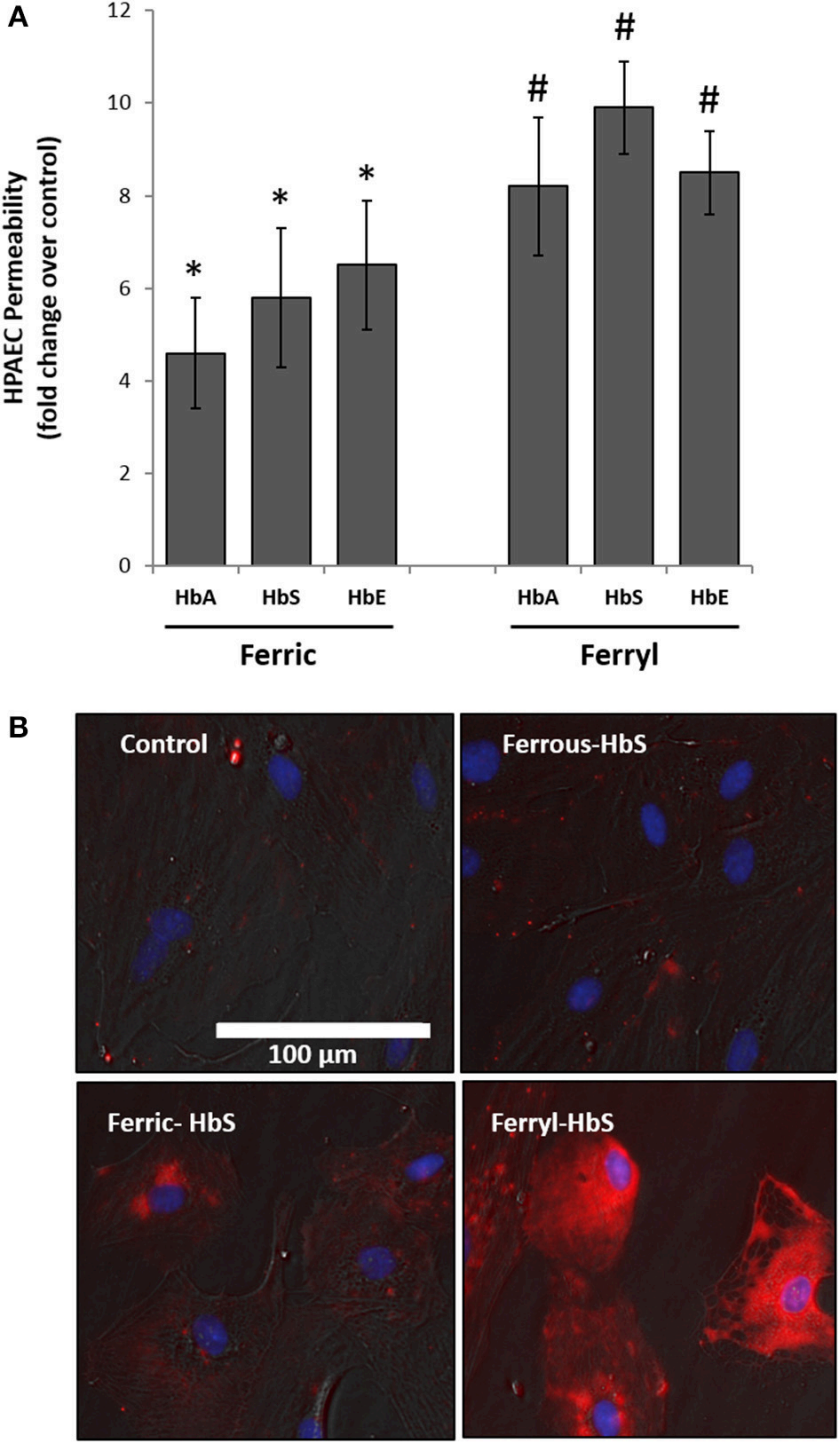
Oxidized hemoglobin variants cause rapid disruption of pulmonary endothelial monolayer and generate oxidative stress. **(A)** HPAEC monolayer were grown on Transwell inserts and then exposed to either ferric or ferryl forms of HbA, HbS, or HbE at equimolar concentration (100 μM) for 6 h in the presence of FITC conjugated dextran, 40 KD. Endothelial monolayer integrity was assessed by measuring the passage of FITC-dextran as mentioned in the methods section. Values are presented as average fold difference over control (*N* = 3). Differences between groups were compared using Mann-Whitney *U*-test. ^*^P < 0.05 and ^#^*P* < 0.001 vs. respective controls. **(B)** HPAEC were exposed to ferryl Hb proteins at equimolar concentration (100 μM) for 6 h. Following incubation cells were washed thoroughly and probed with spin trapping agent DMPO. DMPO-nitrone adducts formed with oxidatively modified proteins within HPAEC were visualized by immunofluorescence method using anti-DMPO-nitrone antibody. Images shown are from one representative set of experiments repeated three times.

To explore further the oxidative toxicity on pulmonary endothelial cells we utilized oxidized forms of individual Hb variants, e.g., ferric and ferryl, for the endothelial permeability experiments. Unlike the ferrous forms all the ferric forms of Hb variants could disrupt the endothelial barrier function to a much greater degree (Figure 4A). Similarly, when the ferryl forms were used, we noticed greater leakage of dextran molecules through the HPAEC monolayer compared to respective ferric Hb treatments within a short period of incubation (6 h).

To assess the extent of oxidative changes within endothelial cells following treatment with higher oxidation Hb, we first loaded cells with DMPO and then used a polyclonal antibody-based immunofluorescence approach directed against multiple epitopes bearing DMPO-nitrone protein adducts. For this purpose, we selected HbS due to its higher oxidative potential as already observed in our earlier published results (Kassa et al., 2015). HPAEC were exposed to either reduced or oxidized (ferric or ferryl) of HbS for 2 h followed by labeling with DMPO. Figure 4B shows a gradual appearance of DMPO-bound protein adducts (labeled as red) with higher oxidation states of Hb reaching maximum with the ferryl state.

### Ferryl hemoglobin causes bioenergetic impairment in HPAEC

**Figure 5:**
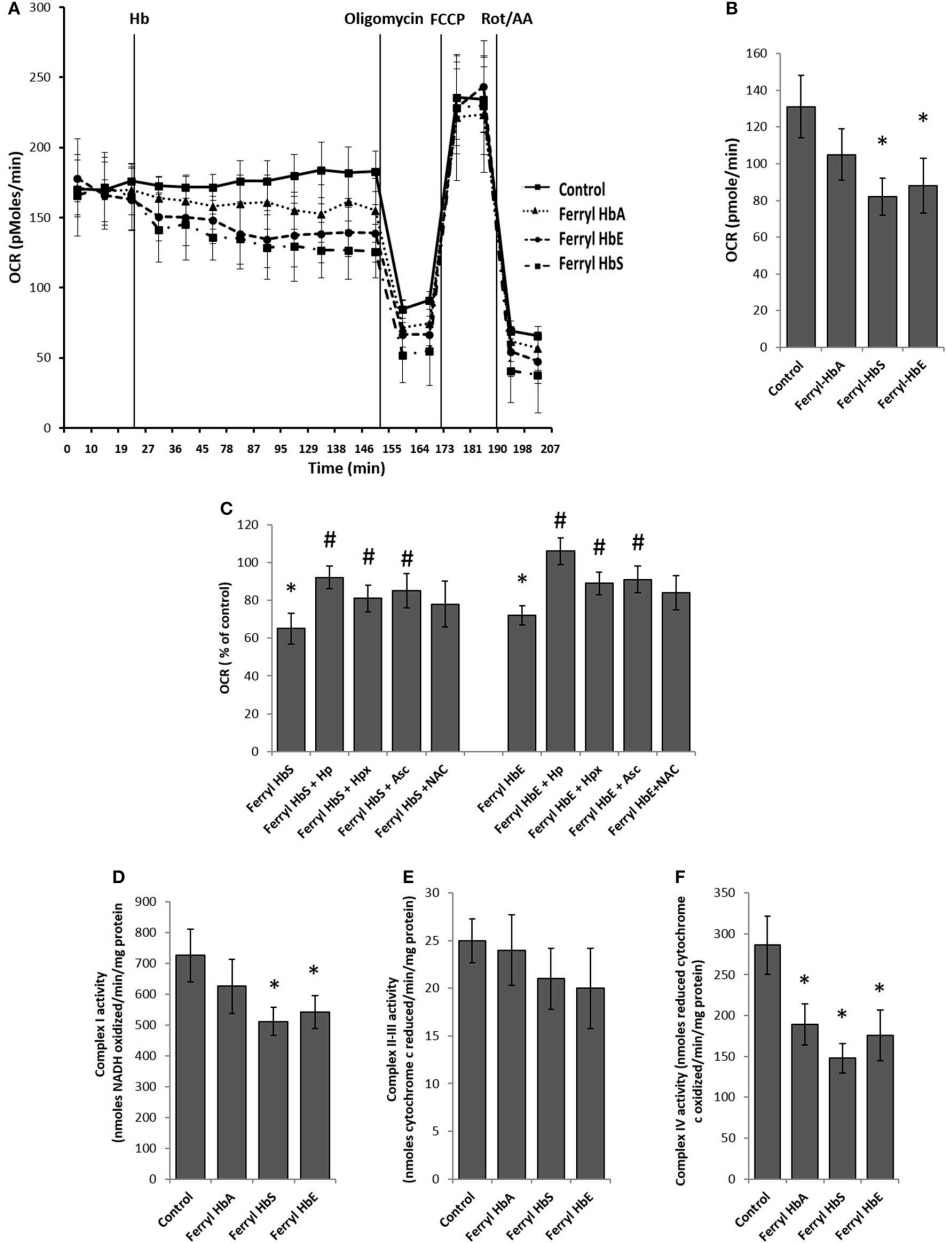
Oxidized ferryl hemoglobins disrupt pulmonary endothelial bioenergetics. **(A)** HPAEC were grown on 24 well XF-plates. Mitochondrial oxygen consumption rates (OCR) were measured for 2 h using XF24 extracellular flux analyzer with direct infusion of ferryl forms of HbA, HbS, or HbE at equimolar concentration (100 μM) (*N* = 3). **(B)** Basal OCR following 2 h incubation with ferryl Hbs (*N* = 3). **(C)** Average basal OCR values from a similar set of experiments in which HPAEC were exposed to either ferryl HbS (100 μM) or ferryl HbE (100 μM) for 2 h co-incubated with or without equimolar Hp or Hpx or Asc or NAC (*N* = 3). Statistical significance between means was calculated by paired Student's *t*-test. ^*^P < 0.05 vs. untreated control, ^#^*P* < 0.05 vs. respective ferryl Hb. **(D)** Complex I activity **(E)** complex II-III and **(F)** complex IV activities were measured in isolated mitochondrial fractions incubated with different ferryl Hb proteins for 2 h at 37°C (*N* = 4). Differences between individual treated groups and respective untreated controls were compared using Mann-Whitney *U*-test. ^*^P < 0.05 vs. controls.

Rapid impairment of endothelial permeability by oxidized species of Hb prompted us to investigate metabolic changes in HPAEC following exposure to ferryl species of the different Hb variants. We carried out bioenergetic measurements in the presence of different ferryl Hb species to assess their impact on endothelial respiration. To achieve this, first we added freshly prepared ferryl Hb variants directly on the loading cartridge of XF-analyzer. Upon injection of ferryl HbA directly into the cell culture media, an insignificant decrease in basal respiration was observed over control cells treated with vehicle. However, a gradual but consistent decrease in basal respiration occurred in HPAECs following addition of equimolar ferryl HbS into the media (Figures 5A,B). A similar bioenergetic pattern was also obtained following a direct addition of ferryl HbE (Figures 5A,B). However, ferryl Hbs did not cause any noticeable changes in respiration in the uncoupled state following FCCP treatment (Figure 5A). We also studied changes in glycolytic rate by monitoring ECAR in HPAEC within short incubation time of 2 h. Ferryl Hbs in a similar experimental set up did not cause any changes in glycolysis within 2 h incubation as evidenced by unaltered ECAR in HPAEC (data not shown).

To understand the role of ferryl Hb in causing endothelial bioenergetic impairment various scavengers and antioxidants were added to the medium with either ferryl HbS or HbE. Haptoglobin (Hp) almost completely abolished ferryl Hb mediated loss of basal respiration whereas heme scavenger Hpx showed only partial protection (Figure 5C). Ascorbate was also able to partially prevent ferryl HbS or ferryl HbE mediated fall of basal OCR. However, ferryl HbA induced loss of basal OCR was not prevented by NAC (Figure 5C). Unlike the general-purpose antioxidant NAC, Asc is known to specifically reduce both ferric and ferryl forms of Hb to their lower redox state (Dunne et al., 2006).

In the presence of substrates electrons shuttle through mitochondrial respiratory complexes by oxidative phosphorylation and represent the basal OCR. Complex V is a point of proton reentry from the mitochondrial intermembrane space to the matrix, and inhibition of this activity could decrease basal OCR and alter membrane potential (Δψ) and ROS generation (Cardenes et al., 2014). To test whether ferryl Hb induces any impairment in the mitochondrial respiratory chain components we first isolated mitochondrial fraction from cultured HPAEC and then exposed mitochondrial fractions to highly reactive ferryl Hb variants for 2 h at 37°C. Following incubation with Hb, individual electron transport chain complexes were assayed using specific substrates and inhibitors as described earlier in the methods. Figure 5D shows that oxidation of reduced NADH by mitochondrial complex I was marginally compromised by ferryl HbS and ferryl HbE but not by ferryl HbA. In contrast, antimycin A sensitive reduction of cytochrome c by complex II–III (succinate–cytochrome c reductase) was not affected by any of the ferryl Hb variants (Figure 5E). However, cyanide sensitive oxidation of ferro-cytochrome c by complex IV (cytochrome c oxidase) was markedly impacted by all the ferryl Hb variants following an incubation of 2 h (Figure 5F).

### Ferryl hemoglobin induces metabolic reprogramming

**Figure 6:**
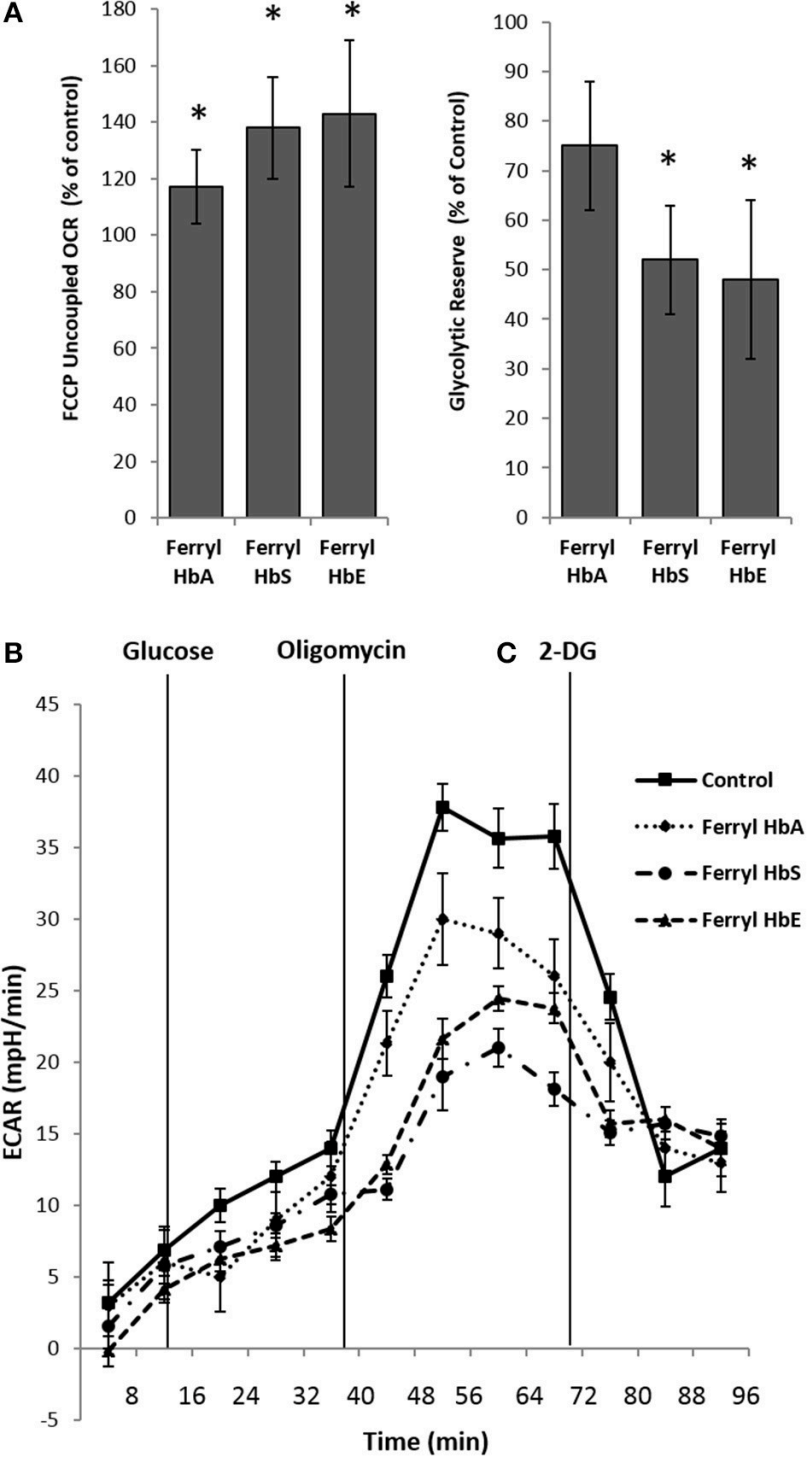
Oxidized hemoglobins cause bioenergetic reprogramming in pulmonary endothelial cells. HPAEC were exposed to ferryl forms of HbA or HbS or HbE at equimolar concentration (100 μM) for 12 h. Mitochondrial oxygen consumption rates (OCR) and extracellular acidification rates (ECAR) were measured in real time using XF24 extracellular flux analyzer. **(A)** Average maximal OCR values expressed as % of control in HPAEC obtained after uncoupling of mitochondria following FCCP treatment (*N* = 3). **(B)** Average glycolytic reserve capacity expressed as (%) of control in HPAEC (*N* = 4) **(C)** representative ECAR plots of ferryl Hb treated HPAEC from an individual set of experiment repeated three times. Statistical significance between means was calculated by paired Student's *t*-test. ^*^P < 0.05 vs. untreated control.

Next we examined the bioenergetic profile of pulmonary endothelium following a prolonged incubation (12 h) with various ferryl species of Hb. Surprisingly, all the ferryl Hb proteins caused a significant increase in FCCP-induced uncoupled respiration (maximal respiration) in HPAEC over untreated control (Figure 6A). In a similar experimental setup, ferryl species of both mutant Hb proteins i.e., ferryl HbS and ferryl HbE significantly impacted glycolytic rates (ECAR) within HPAECs following addition of glucose (Figures 6B,C). Figure 6B highlights the fall in glycolytic reserve in HPAECs induced by ferryl HbS and ferryl HbE.

## Discussion

The primary aim of this study was to evaluate the unique oxidative pathways of HbS and HbE and their impact on human pulmonary arterial endothelial cells (HPAEC) and to compare that to normal HbA. Cell free plasma Hb, and its prosthetic heme, are known to disrupt endothelial function, drive oxidative and inflammatory stress. Heme, the byproduct of Hb oxidation is also recognized as a damage-associated molecular pattern (DAMP) molecule (Gladwin et al., 2010). Studies in a sickle cell mice model showed unequivocally that heme released from plasma oxidized Hb binds to the toll-like receptor-4 (TLR-4) on macrophages that can activate the innate immune system (Belcher et al., 2014). Persistent intravascular hemolysis in SCD leads, over decades, to chronic vasculopathy, with some ~10% of patients developing pulmonary hypertension (Potoka and Gladwin, 2015). Adult and children with HbE-β thalassemia are also reported to present vascular complications as a result of the oxidative stress experienced by these patients (Kukongviriyapan et al., 2008). In both diseases, RBC-derived microparticles were shown to promote vascular complications as they carry both highly oxidized Hb and considerable quantities of free heme to the vascular system (Chaichompoo et al., 2012; Camus et al., 2015)

Naturally occurring human Hb variants (resulting mostly from single point mutations) alter Hb structure and biochemical properties with physiological effects ranging from insignificant to severe (Thom et al., 2013). These Hb mutants, which are subjected to evolutionary pressures, can provide unique model systems to address the question as to why some Hb variants are more oxidatively stable while others succumb to oxidative stress and develop into a full circulatory disorder (Thom et al., 2013). An example of an oxidatively stable Hb mutant we reported recently is Hb Providence, in which two mutations exist in these patient RBCs: βLys82-Asp (βK82D) and at βLys82-Asn (βK82N) positions; both of these fractions resist H_2_O_2_ induced oxidation by internalizing radicals through the ferric/ferryl pseudoperoxidase cycle (Abraham et al., 2011). Conversely, single amino acid variant Hbs linked to severe pathology, including sickle cell Hb (HbS) (β6 Glu-Val) and HbE (β26 Glu-Lys), are less oxidatively stable than HbA. We have recently found that both ferryl forms and associated radicals of HbS and HbE persist longer *in vitro* than their HbA counterpart by targeting and specifically oxidizing βCys93 which subsequently leads to heme loss from the proteins (Kassa et al., 2015; Strader et al., 2016).

To assess the role of HbS and HbE in promoting endothelial functional impairment, we monitored endothelial permeability, intracellular oxidative stress response parameters and some key metabolic changes involving mitochondrial bioenergetics and changes in glycolytic flux. Vascular endothelium very selectively allows passage of blood or plasma components into the adjacent tissues by acting as a dynamic barrier. However, vascular endothelial integrity is highly susceptible to damage by various stress conditions including production of inflammatory mediators (Nooteboom et al., 2000). The passage of FITC-conjugated 10KD dextran molecules through HPAEC monolayers grown on Transwell inserts used in our investigation clearly demonstrated the impairment of membrane integrity by HbS and HbE, but not by HbA, during a prolonged incubation with a concomitant rise in intracellular oxidative stress response in HPAEC. This phenomenon may be attributed to the previously documented higher rates of autoxidation, oxidative instability and heme release from the two mutant Hb proteins compared to common HbA (Table 1). Higher rates of autoxidation of mutant Hbs coupled with the formation of more persistent and damaging ferryl species under oxidative stress conditions can target other biological molecules (Kassa et al., 2015). We have recently shown that ferric Hb loses heme at rates substantially higher than that of ferryl Hb (Kassa et al., 2016). This was also supported by a higher expression of heme oxygenase-1 (HO-1) when ferric Hb was added to cultured lung alveolar epithelial cells (E10). The mitochondrial dysfunction reported here may therefore be attributed to a combination of accelerated heme loss from the ferric form and protein radical formation associated with the ferryl Hb. This is consistent with a recent study in which it was shown that ferric (metHb) causes oxidative damage to myelin basic protein (MBP) and myelin lipids, partly by transferring its heme moiety to protein and lipid, but mostly as an intact protein possibly via formation of a ferryl radical (Bamm et al., 2017). Our finding of high levels of DMPO-bound protein radicals within ferryl Hb treated HPAECs supports this possibility. Interestingly, we also noticed a high degree of HPAEC membrane leakage mediated by the oxidized Hb molecules; this finding is consistent with a recent observation where a greater loss of endothelial monolayer integrity was evident under ferryl Hb exposure (Lisk et al., 2013). The resulting endothelial damage was largely attributed to the MyD88 mediated mechanism involving NF-κB and HIF-1α activation. Our results further show that mutant Hb proteins, e.g., HbS and HbE, due to their distinct oxidative profile can elicit a more profound oxidative stress response than common HbA in human endothelial cells through upregulation of HO-1, ferritin and activation of NF-κB. Findings from another previous work showing differential reactivity of these mutant Hbs causing lipid peroxidation also strongly supports our results (Chiu et al., 1996). These events can be attributed to their heme releasing capability in addition to their high redox potential (Bonaventura et al., 2002; Roche et al., 2011).

Studies on endothelial energy metabolism have shown the dominance of glycolysis over mitochondrial ATP production, although endothelial mitochondria are considered critical for the maintenance of functional integrity of these special vascular cells (Davidson and Duchen, 2007; Groschner et al., 2012). Endothelial mitochondria can integrate several cell death and survival pathways through transition pore opening, intracellular calcium signaling and redox signaling (Davidson and Duchen, 2007). Growing evidence supports the notion that endothelial mitochondria may actually act as a sensor for oxygen and for the vasodilatory response of endothelial nitric oxide and thus can play a “reconnaissance” role through relaying information to adjacent cardiac myocytes or smooth muscle cells (Davidson and Duchen, 2007). Our present study is undoubtedly the first to observe bioenergetic changes in human endothelial cells mediated by the Hb variants. Since, endothelial cells are more dependent on glycolytic ATP production, we also for the first time monitored changes in glycolytic rates under the influence of Hb exposure. Despite some early signs of leakage in endothelial monolayers, neither of the Hb variants in reduced states showed any imbalance in basal respiration or glycolysis in HPAECs. However, an augmentation of uncoupled respiration by both HbS and HbE associated with more mitochondrial oxygen radical production and lipid peroxidation supports a mechanism linked to higher rates of oxidation and greater heme release by the two proteins. Carbon monoxide is one of the major byproduct of heme catabolism by HO-1 and is known to cause inhibition of glycolysis and uncoupling of mitochondrial respiration by opening mitochondria specific ion-channels in endothelium (Kaczara et al., 2015, 2016).

Our results clearly show that direct interactions of oxidized Hbs with vascular endothelium can cause severe impairment of selective electron transport chain complexes especially cytochrome c oxidase. This explains the ferryl Hb mediated loss of mitochondrial function in intact endothelial cells during a short co-incubation. In contrast, all the ferryl Hbs also augmented uncoupled respiration quite significantly after a longer incubation. We have previously shown that HO-1 is strongly expressed by both ferric and ferryl Hbs (Chintagari et al., 2016; Kassa et al., 2016). Therefore, a boost in maximal respiration following uncoupling by FCCP can be easily correlated with our finding that Hb proteins promote high expression of stress response proteins in varying degree depending on their oxidative profile and possibly be considered as an outcome of HO-1 mediated CO production. A concomitant loss of glycolytic reserve in HPAEC by ferryl Hbs after a long incubation also indicates a metabolic reprogramming in the endothelium probably due to an exhaustive loss of glycolytic capacity in HPAEC. Similar observations have been seen in some cancer cells where a prolonged uncoupled state of mitochondrial respiration resulted in metabolic exhaustion and loss of glycolysis (Wegiel et al., 2013).

In summary, we show for the first time a comparative toxicity of different mutant Hbs on a pulmonary endothelial model based on their distinct oxidative profile; and also propose a mechanism that explains Hb-induced metabolic and functional alteration. Our results elucidating the mechanism of extracellular Hb-induced oxidative damage to the mitochondrial respiratory chain will contribute to better understanding of some of the underlying pathophysiology of pulmonary arterial hypertension caused by cell free Hbs; and, in addition, lend new insight into potential antioxidative therapeutic interventions in hemolytic diseases.

## Author contributions

Conception and design: SJ and AA. Experiments: SJ and FM. Data Analysis: SJ and FM. Drafting manuscript: SJ, RH, JF, and AA.

### Conflict of interest statement

The authors declare that the research was conducted in the absence of any commercial or financial relationships that could be construed as a potential conflict of interest.
